# Adaptation of rhizosphere bacterial communities of drought-resistant sugarcane varieties under different degrees of drought stress

**DOI:** 10.1128/spectrum.01184-23

**Published:** 2023-09-12

**Authors:** Jicao Dao, Yuanjun Xing, Chunyi Chen, Mianhe Chen, Ziting Wang

**Affiliations:** 1 Guangxi Key Laboratory of Sugarcane Biology, Nanning, Guangxi, China; 2 State Key Laboratory for Conservation & Utilization of Subtropical Agro-bioresources, Guangxi University, Nanning, Guangxi, China; 3 College of Agronomy, Guangxi University, Nanning, Guangxi, China; Instituto de Ecología A.C., Pátzcuaro, Michoacán, Mexico

**Keywords:** drought gradient, sugarcane, rhizosphere bacterial community, core functional bacteria, drought response

## Abstract

Sugarcane is highly sensitive to changes in moisture, and increased drought severely restricts its growth and productivity. Recent studies have shown that plant growth-promoting microorganisms are essential to reduce the adverse effects of environmental stresses, especially drought. However, our knowledge about the dynamics of rhizosphere microbial community structure in sugarcane under varying degrees of drought stress is limited. We analyzed the effects of different degrees of drought stress on the rhizosphere microbial communities of Zhongzhe 1(ZZ1) and Zhongzhe 6(ZZ6) with differences in drought resistance, by combining soil enzyme activity, nutrient content, and physiological and morphological characteristics of sugarcane roots. The results showed that rhizosphere bacterial community began to change at a field capacity of 50%, enriching the sugarcane rhizosphere with drought-resistant bacteria. The core strains of ZZ1 and ZZ6 rhizosphere enrichment were mainly *Streptomycetales*, *Sphingomonadales*, and *Rhizobiales*. However, compared to ZZ1, the changes in rhizosphere bacterial abundance in ZZ6 were primarily associated with the abundance of *Streptomycetales* as drought levels increased. *Rhizobiales* and *Streptomycetales*, enriched in the rhizosphere of ZZ6 under drought, were positively correlated with root tip number and total root length (TRL), increasing the distribution area of roots and, thus, improving water and nutrient uptake by the roots thereby enhancing the resistance of sugarcane to drought stress. This research enhances our understanding of the composition of the rhizosphere microbial community in sugarcane under different levels of drought stress and its interaction with the roots, thereby providing valuable insights for enhancing drought resistance in sugarcane.

**Importance:**

Drought stress is expected to further increase in intensity, frequency, and duration, causing substantial losses in sugarcane yields. Here, we exposed sugarcane to varying degrees of drought treatment during growth and quantified the eventual composition of the resulting sugarcane rhizosphere bacterial community groups. We found that sugarcane rhizosphere under mild drought began to recruit specific bacterial communities to resist drought stress and used the interactions of root tip number, total root length, and drought-resistant strains to improve sugarcane survival under drought. This research provides a theoretical basis for the rhizosphere microbiome to help sugarcane improve its resistance under different levels of drought stress.

## INTRODUCTION

Global climate change has led to an abrupt increase in the frequency of drought stress through increased temperatures and altered precipitation patterns, which are the leading causes of potential crop yield declines (1, 2). Although plant adaptation to drought stress is a complex process, drought stress and the reduction in plant soil water reduce nutrient availability through mineralization, which influences morphological, physiological-biochemical, and metabolic responses that affect plant growth, development, and survival (3 – 5). In particular, root systems, such as plants subjected to drought stress, increase their ability to access soil water by increasing root length and surface area (SA) (6, 7). Sugarcane (*Saccharum* spp.) is highly sensitive to water deficits during growth, and an excessive water deficit can slow stalk biomass accumulation, resulting in substantial yield losses (8, 9). Previous studies have found that drought stress can limit sugarcane growth by dwarfing plants and limiting tillering, ultimately causing an overall decrease in the yield attributes and sugarcane juice quality (9 – 11). Therefore, strengthening the body of research on sugarcane responses to drought stress is an effective method to ensure a stable yield of sugar crops against drought.

Sugarcane is a globally important cash crop widely used in energy, ethanol, and sugar production (12, 13). One of the main characteristics of sugarcane production is high water demand during the growth phase. In recent years, the uneven distribution of precipitation in growing areas has exposed sugarcane to frequent water-deficit environments, with significant effects on its morphological, physiological, and biochemical characteristics (13, 14). Current research indicates that timely regulation when drought levels fail to cause irreversible damage to the crop can lead to normal growth and development (15, 16); therefore, investigating the stress response of sugarcane to drought stress is necessary to improve its drought tolerance. Under water-deficit conditions, sugarcane improves drought tolerance by changing its physiological characteristics and root traits (17, 18). Moreover, owing to genetic differences, plant growth traits are highly diverse in response to drought stress (19), and such differences are reflected among varieties (20). For instance, the Zhongzhe series of sugarcane varieties exhibit advantages such as high yield, high sugar content, and good lodging resistance compared to other varieties. Furthermore, this variety can reduce damage to the plant itself by increasing superoxide dismutase activity to scavenge reactive oxygen species produced by drought stress (21, 22). Previous studies have shown that the drought resistance of Zhongzhe series varieties is relatively strong, with ZZ6 outperforming ZZ1 based on average affiliation function values (23). Indole-3-acetic acid (IAA)-producing bacteria enriched in the sugarcane rhizosphere under drought conditions alleviate drought stress in plants by stimulating plant growth and root elongation, allowing sugarcane to obtain more nutrients and water from deeper soils (24, 25). Therefore, investigating the response mechanisms of sugarcane under drought conditions, the selection and breeding of superior drought-resistant varieties, and the association mechanisms between the sugarcane root system and rhizosphere microorganisms are crucial to support healthy plant growth and ensure yield under drought conditions.

Various microorganisms inhabit different parts of sugarcane, including leaves, stalks, roots, and the rhizosphere. These microorganisms play a crucial role in plant adaptation to various types of stress, such as water stress, pathogen attacks, and nutrient deficiencies (26, 27). In the rhizosphere, plant-microbe interactions are effective in aiding crop development and mitigating the negative effects of drought on crops through nitrogen fixation, nutrient acquisition by antimicrobial metabolites, phosphorus solubilization, binding of microbial biofilms to plant roots, and induction of phytohormone production (28 – 31). Previous studies have demonstrated that drought stress significantly reduces the diversity of rhizosphere bacteria in sugarcane and increases the abundance of drought-tolerant bacteria (32). Changes in the abundance of rhizosphere bacteria under drought stress primarily occur within the phyla Rhizobium and Streptomyces (33). *Rhizobiales* can form beneficial associations with plants to fix nitrogen, provide nutrients, or produce phytohormones (34, 35). Similarly, actinomycetes can enter a steady state during stress because of their sporulation capacity and induce the production of plant growth hormones, such as IAA and gibberellic acid, which allow them to survive under drought and assist plants in resisting drought (36, 37). Previous studies have shown that drought tolerance in sugarcane can be affected by a combination of rhizosphere drought-tolerant bacteria and root exudates (32).

Plants possess the ability to selectively colonize their rhizosphere with microorganisms, and root exudates play a crucial role in shaping the rhizosphere microbiome (38 – 40). An increase in the organic acid exudation rate of sugarcane plants under drought conditions may result in the formation of diverse communities with unique characteristics, thus mitigating the effects of low soil moisture and improving crop tolerance to drought (41, 42). Most sugarcane research has focused on improving sugar yield and breeding varieties resistant to adverse stress, as well as on sugarcane rhizosphere microorganisms that assist plants in resisting drought (27, 43). However, a large cognitive gap remains in the performance of sugarcane rhizosphere microorganisms in terms of stress tolerance under different drought intensities. The close interaction between plants and their associated microbiota can influence the health of the host plant and its response to environmental changes (44). Despite advancements in the study of sugarcane rhizosphere microbiota, the role of sugarcane species in regulating this community under different drought intensities remains poorly understood. Therefore, to comprehend how rhizosphere microorganisms assist plants in resisting stress under drought conditions, it is necessary to explore changes in the response of sugarcane rhizosphere microorganisms in addition to studying changes in the core flora.

In this study, we conducted 16S rRNA sequencing analysis to investigate the differential changes in rhizosphere microbial communities of two sugarcane species (ZZ1 and ZZ6) under different levels of drought stress. Our study aimed to investigate two questions: (1) How does the composition of the rhizosphere bacterial community vary among sugarcane cultivars under a drought gradient? (2) How do rhizosphere core drought-resistant bacteria from different sugarcane varieties respond to stress and help plants resist drought under a drought gradient?

## RESULTS

### Correlation of root system characteristics with soil physicochemical properties and aboveground physiological characteristics

**Fig 1:**
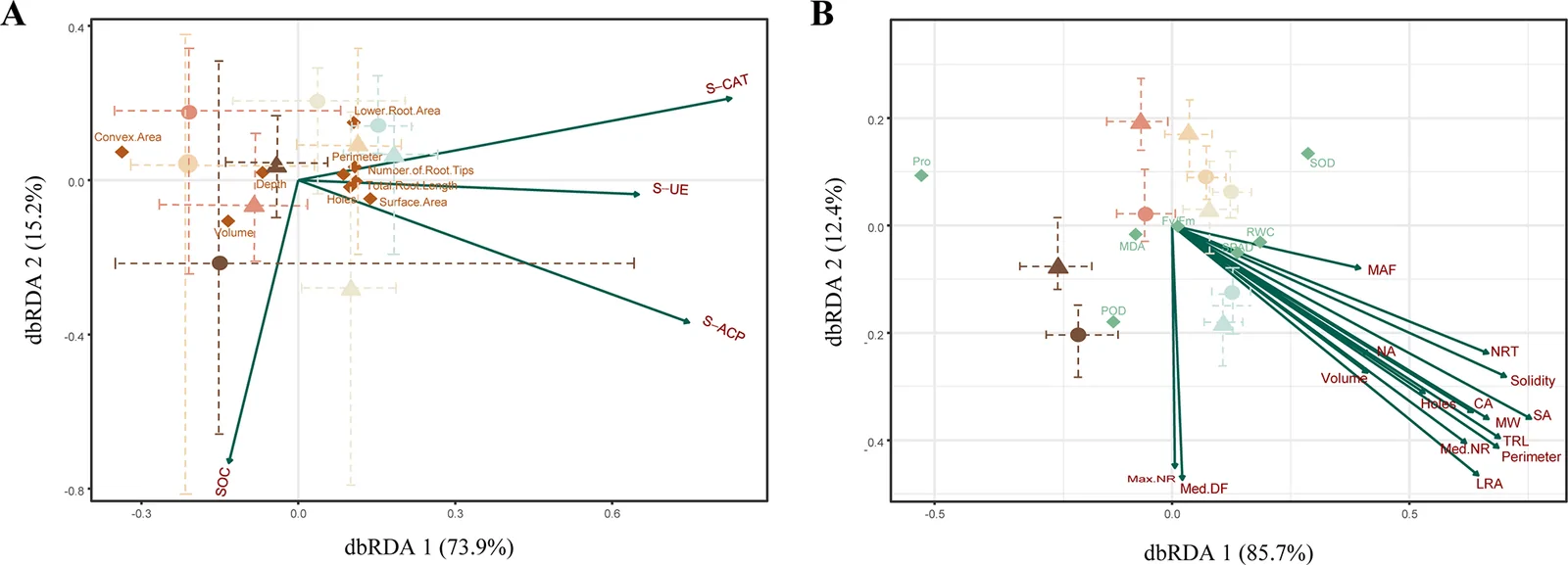
The dbRDA analysis of root traits, soil physicochemical properties, and environmental factors. (**A**) The dbRDA analysis of relationship between environmental factors and root traits and soil physicochemical properties in five water stress patterns. (B) The dbRDA analysis of relationship between the root and aboveground physiological characteristics in five water stress patterns. Full abbreviation: MAF, medium angle frequency; NRT, number of root tips; SA, surface area; NA, network area; CA, convex area; MW, maximum width; TRL, total root length; Med.NR, median number of roots; Med.DF, medium diameter frequency; Max.NR, maximum number of roots.

We utilized distance-based redundancy analysis (dbRDA) to investigate the relationship between environmental factors, root characteristics, and soil physicochemical properties across five water stress patterns. Our analysis revealed that changes in root characteristics had a substantial impact on soil physicochemical properties, and this association was observed across different levels of drought. By incorporating drought stress gradients and varietal differences as primary explanatory variables, we found that water stress accounted for 89.1% (dbRDA1: 73.9%, dbRDA2: 15.2%) of the correlation between sugarcane root architecture and soil physicochemical properties (Fig. 1A). Compared with other characteristics, the indicator arrows represented by catalase (S-CAT), soil acid phosphatase (S-ACP), and soil organic carbon (SOC) in Fig. 1 focus on areas where root characteristics, such as the number of root tips (NRT) and root SA, were majorly clustered, indicating a strong correlation between S-CAT, S-ACP, SOC, and sugarcane root conformation (longer line arrows) (Fig. 1A). We further explored the relationship between root characteristics and aboveground plant properties. The dbRDA analyzed the relationship between the root and aboveground physiological characteristics, with the first two axes accounting for 85.7% and 12.4% of the total data variance, respectively (Fig. 1B). There was some overlap or slight angle between the characteristic indicators of root conformation and different aboveground physicochemical properties. Notably, NRT was strongly influenced by chlorophyll index (SPAD) and leaf relative water content, while medium diameter frequency exhibited a high correlation with maximum quantum yield of Photosystem II (Fv/Fm), malondialdehyde (MDA), and peroxidase (Fig. 1B). Additionally, the degree of drought stress significantly affected the aggregation of the samples. Root characteristics such as SA, lower root area (LRA), total root length (TRL), medium diameter frequency, and perimeter-affected field capacity (FC) 20, FC30, CK, FC50, and FC40 were distributed on the left side of the dbRDA2 axis, indicating that aboveground physiological characteristics were significantly affected by drought stress (Fig. 1B).

### Correlation of rhizosphere bacterial diversity and root morphology under drought gradients

**Fig 2:**
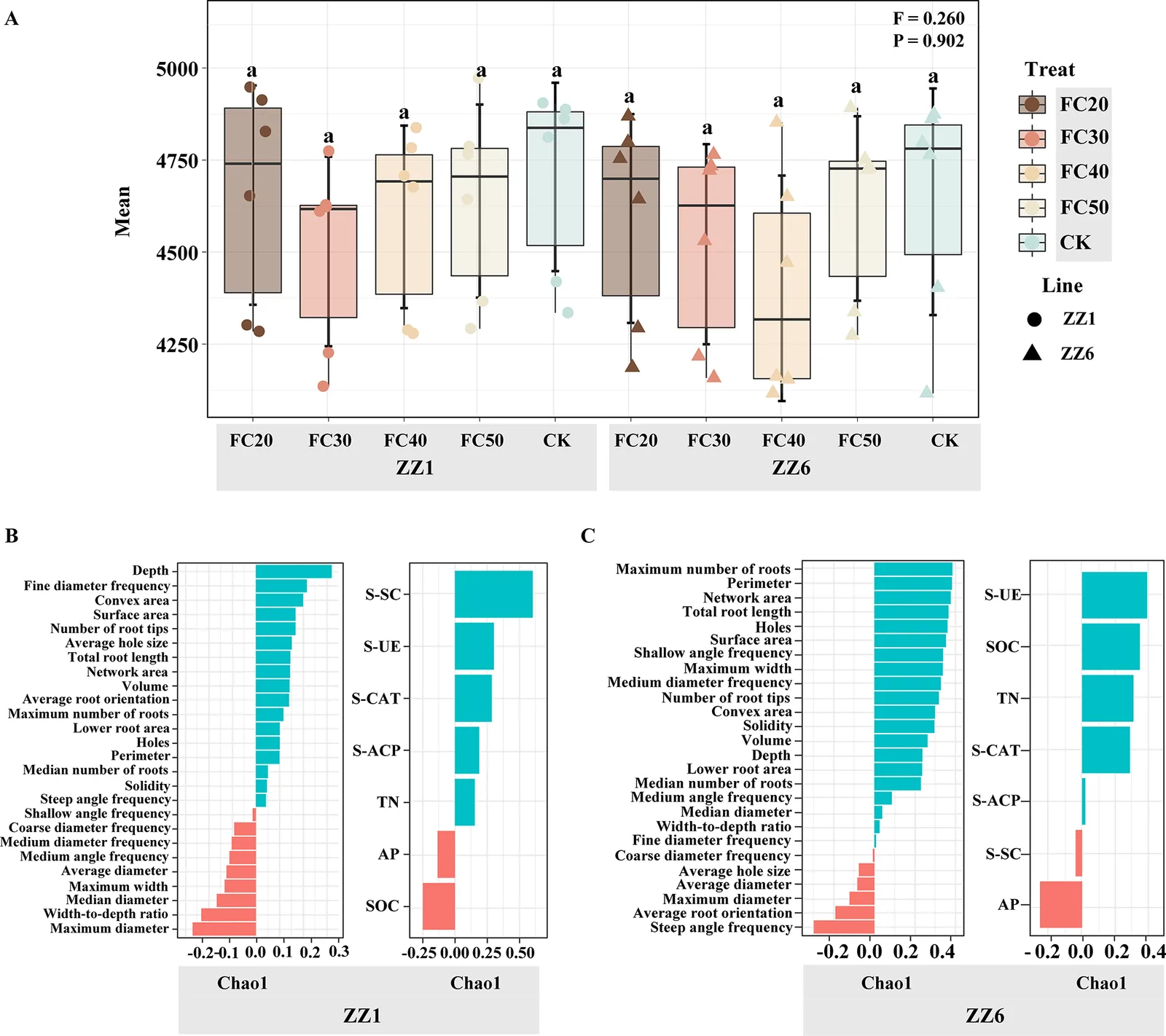
Differential analysis of microbial diversity based on drought gradient treatments and varietal differences-Chao1 index analysis. (**A**) Chao 1 abundance among different drought gradient treatments and varieties. (**B**) Pearson correlation analysis of Chao 1 index with root traits and soil physicochemical properties for ZZ1 varieties, with positive correlation in blue and negative correlation in red. (**C**) Pearson correlation analysis of Chao 1 index of ZZ6 varieties with root traits and soil physicochemical properties, blue color shows positive correlation and red color shows negative correlation.

**Fig 3:**
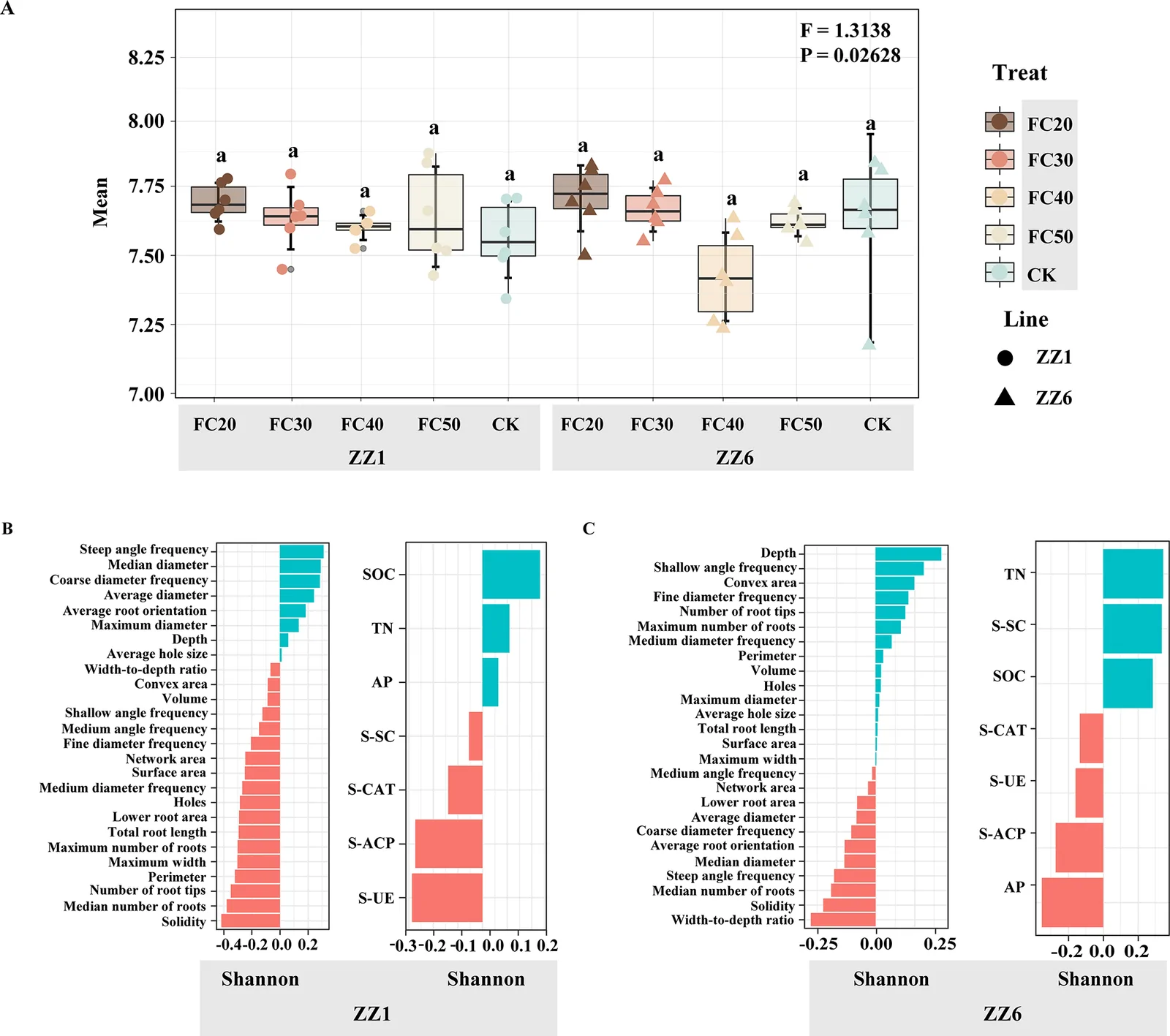
Differential analysis of microbial diversity based on drought gradient treatments and varietal differences—Shannon index analysis. (**A**) Shannon abundance among different drought gradient treatments and varieties. (**B and C**) Pearson correlation analysis of Shannon index with root traits and soil physicochemical properties for different varieties, blue shows positive correlation and red shows negative correlation.

Analysis of the 16S rRNA sequencing data of rhizosphere bacteria under drought stress revealed no significant differences in the α-diversity of rhizosphere bacterial communities across different drought stress gradients, and the variations between the two sugarcane varieties were not statistically significant (Fig. 2 and 3). From the overall trend of α-diversity of the sugarcane rhizosphere bacterial community under different drought gradients, the lowest value of the Chao1 index appeared at 30%–40% of field capacity. The lowest field capacity of the Chao1 index of the two sugarcane varieties differed slightly, with ZZ1 having the lowest rhizosphere bacterial Chao1 index at FC30 and ZZ6 having the lowest value at FC40 (Fig. 2A). The Chao1 index of rhizosphere bacterial diversity in both ZZ1 and ZZ6 displayed similarities in the CK and FC20 treatments (Fig. 2A). We also examined the correlation between species richness of the rhizosphere bacterial community and sugarcane root traits, as well as rhizosphere soil physicochemical properties. Our findings revealed significant correlations between several sugarcane root traits (TRL, perimeter) and soil properties (S-SC, enzyme activities) with the richness of the rhizosphere bacterial community (Fig. 2B and C). However, these correlations varied among species. The correlation between root characteristics and rhizosphere bacterial abundance was not significant in ZZ1, whereas S-SC was significantly positively correlated with rhizosphere bacterial abundance in the soil (Fig. 2B). The maximum number of roots, TRL, network area (NA), perimeter, and holes were significantly and positively correlated with rhizosphere bacterial abundance in ZZ6 (Fig. 2C). In contrast, only soil urease (S-UE) was significantly and positively correlated with rhizosphere bacterial abundance in soil characteristics (Fig. 2C). The Shannon index also showed that the rhizosphere bacterial community did not differ significantly along the drought stress gradient or between the two sugarcane varieties (Fig. 3A). In contrast, the correlation between the Shannon index and root characteristics differed significantly between the two varieties. The Shannon index was mostly negatively correlated with the root characteristics in ZZ1 (Fig. 3B). In contrast, it was mostly positively correlated with the root characteristics in ZZ6 (Fig. 3C). The available phosphorus (AP) positively correlated with the Shannon index in ZZ1 and negatively correlated with the Shannon index in ZZ6 (Fig. 3C).

**Fig 4:**
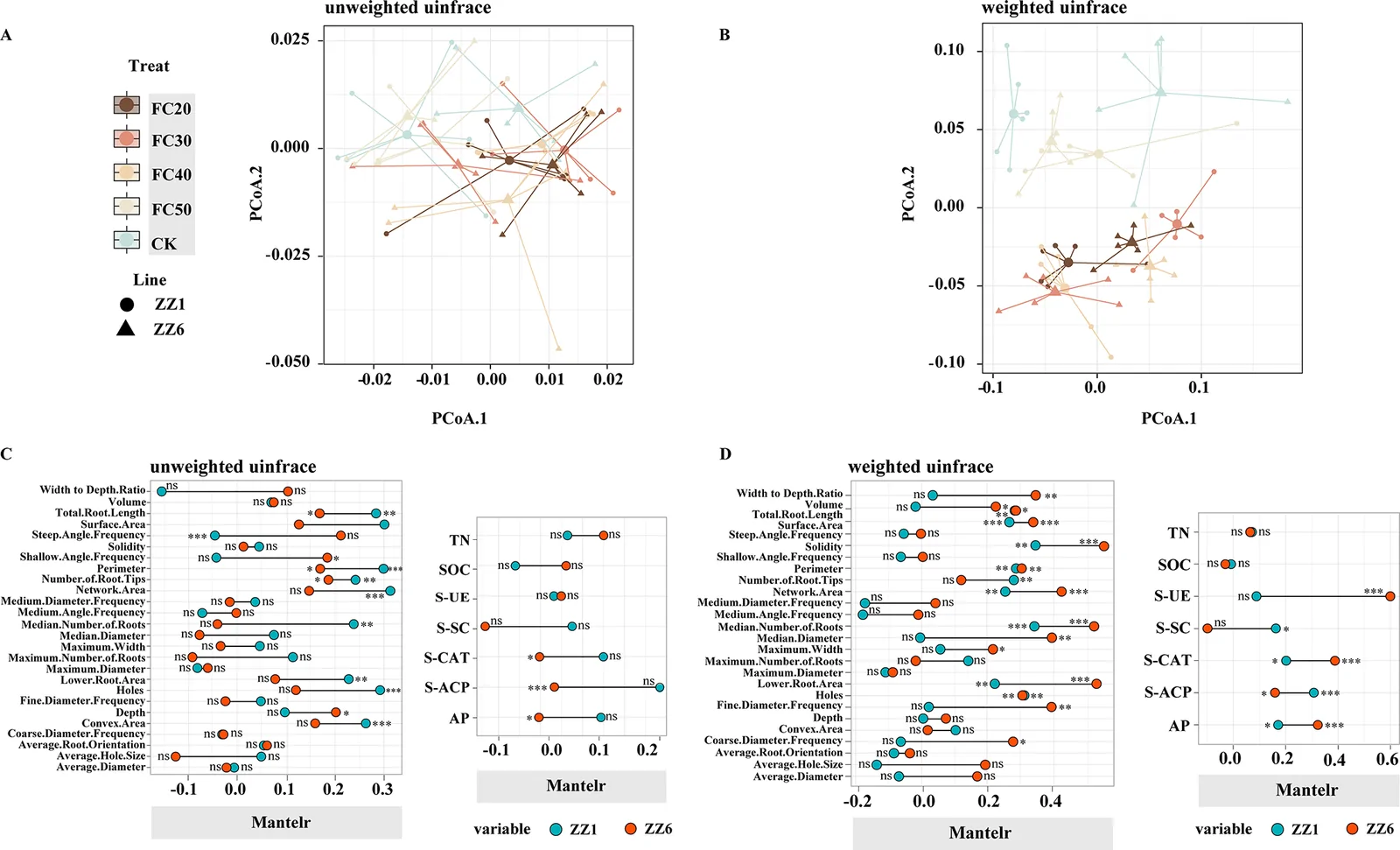
Beta diversity analysis based on drought gradient treatments and varietal differences. (**A and B**) Principal coordinate analysis based on Unifrac distance matrix showing differences in bacterial β-diversity under different drought gradients and species, (**A**) unweighted and (**B**) weighted. Mantel test was used in (**C**) UnifracMantelr and (**D**) weight UnifracMantel analysis to compare the correlated differences in root traits and soil nutrients between ZZ1 and ZZ9. *P < 0.05, **P < 0.01, and ***P < 0.001.

In terms of β-diversity, principal coordinate analysis (PCoA) based on weighted and unweighted UniFrac distances was performed on the rhizosphere bacterial communities in different treatments (Fig. 4A). The PCoA with unweighted and weighted UniFrac distances revealed significant differences among the drought treatments and varieties. Under unweighted conditions, the community composition distribution was relatively concentrated among the drought treatments. Differences in the effects of drought and species on bacterial community composition were not significant (Fig. 4A). Under weighted UniFrac, considering the species partitioning effect, the bacterial community composition differed significantly in the drought treatment, with FC20, FC30, and FC40 distributed on both sides of the first principal axis (PCoA.1) with FC50 and CK (Fig. 4B). The Mantel experiment results showed that the β-diversity of rhizosphere bacteria under drought stress was correlated with the soil physicochemical properties and root characteristics (Fig. 4C and D). According to the Mantel test under unweighted conditions, ZZ1 was significantly correlated (*P* < 0.05) with TRL, SA, steep angle frequency, perimeter, NRT, NA, median number of roots (Med.NR), LRA, holes, and convex area (CA) in terms of root characteristics (Fig. 4C). ZZ6 showed significant correlations (*P* < 0.05) with TRL, steep angle frequency, perimeter, NRT, and depth (Fig. 4C). For soil physicochemical properties, ZZ1 was not significantly correlated with soil physicochemical properties, whereas ZZ6 was significantly correlated with S-CAT, S-ACP, and AP (Fig. 4C). After being weighted, in terms of root characteristics, ZZ1 was significantly correlated (*P* < 0.05) with TRL, SA, solidity, perimeter, NRT, NA, Med.NR, LRA, holes, and fine-diameter frequency. ZZ6 showed significant correlations (*P* < 0.05) with width-to-depth ratio, root volume, TRL, SA, solidity, perimeter, NRT, NA, and Med.NR (Fig. 4D). Regarding soil physicochemical properties, ZZ1 was significantly correlated with S-SC, S-CAT, S-ACP, and AP, whereas ZZ6 was significantly correlated with S-UE, S-CAT, S-ACP, and AP (Fig. 4D).

### Differences in the community composition of rhizosphere bacteria under different stress gradients

**Fig 5:**
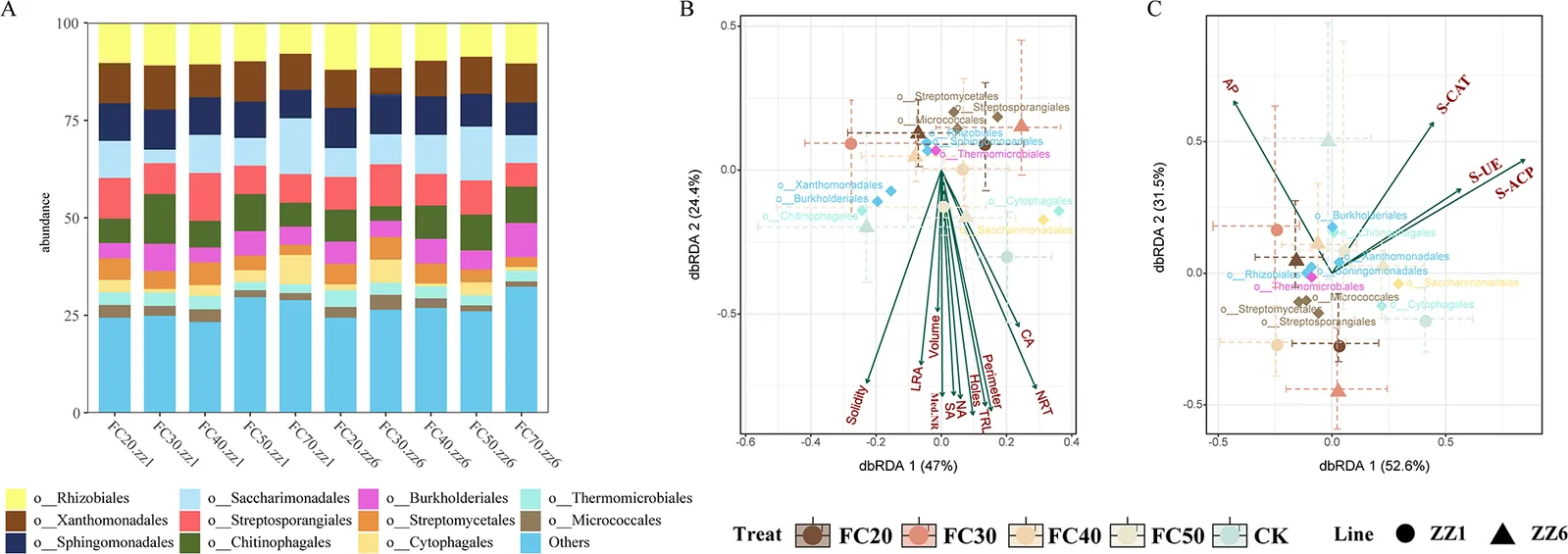
Relative abundance of bacterial species in the rhizosphere bacterial community and their dbRDA redundancy analysis with root traits and soil physicochemical properties. (**A**) Relative abundance of the dominant rhizosphere bacterial community in different treatments. (**B**) Distance redundancy analysis of different models, abundant classes, and root traits (arrows) indicating dominant communities and influencing environmental factors. (**C**) Distance redundancy analysis of different patterns, abundant classes, and soil physicochemical properties (arrows) indicating dominant communities and influencing environmental factors (CA, convex area; NRT, number of root tips; TRL, total root length; NA, network area; SA, surface area; Med.NR, median number of roots; LRA, lower root area).

Class-based population structure analysis of rhizosphere bacteria under different treatments revealed dominant microbial communities in the rhizosphere environment based on their relative abundance. The main groups of sugarcane rhizosphere bacteria were *Rhizobiales*, *Xanthomonadales*, *Sphingomonadales*, *Saccharimonadales*, *Streptosporangiales*, *Chitinophagales*, *Cytophagales*, and *Streptomycetales* (Fig. 5A). The rhizosphere bacterial compositions were similar among different sugarcane varieties, but variations were observed under different drought gradient treatments (Fig. 5A). The species compositions of rhizosphere bacterial communities in ZZ1 and ZZ6 were comparable to those in the control. However, differences were present in the abundances of different bacterial groups. Under ZZ1, the relative abundance of *Streptosporangiales* increased under both FC40 and FC20 compared with the other three moisture treatments (Fig. 5A). However, the relative abundance of *Chitinophagales* increased significantly under FC30. For ZZ6, compared to the control treatments, the relative abundance of *Streptomycetales* and *Saccharimonadales* increased under all four drought treatments, whereas the relative abundance of *Chitinophthora* decreased under FC30 (Fig. 5A).

We further explored the correlation between environmental factors and each component (root characteristics and soil physicochemical properties) in the five water stress patterns using dbRDA. Among the root characteristics for the five water stress patterns, the first two axes of dbRDA accounted for 47% and 24.4% of the total variation, respectively (Fig. 5B). Among these, NRT, TRL, holes, perimeter, and solidity were highly correlated with the composition of sugarcane rhizosphere bacteria. The control (FC70), mild drought (FC50), and moderate and severe drought (FC40, 30, and 20) colony operational taxonomic units (OTUs) were distributed on both sides of the dbRDA2 axis and were significantly affected by drought. *Streptomycetales* and *Streptosporangiales* were mainly distributed in the moderately to severely arid zone, whereas *Chitinophagales* and *Saccharimonadales* were primarily distributed in the mildly arid zone (Fig. 5B). Regarding soil physicochemical properties, the first two axes of dbRDA accounted for 52.6% and 31.5% of the total variation, respectively (Fig. 5C). S-ACP and S-UE were associated with the distribution of bacterial communities in the FC50-ZZ1 and FC50-ZZ6 cells (Fig. 5C). In addition, S-ACP is associated with *Sphingomonadales* and *Saccharimonadales*.

### Changes in the responses of the primary variable strains to water stress and their correlation with roots

**Fig 6:**
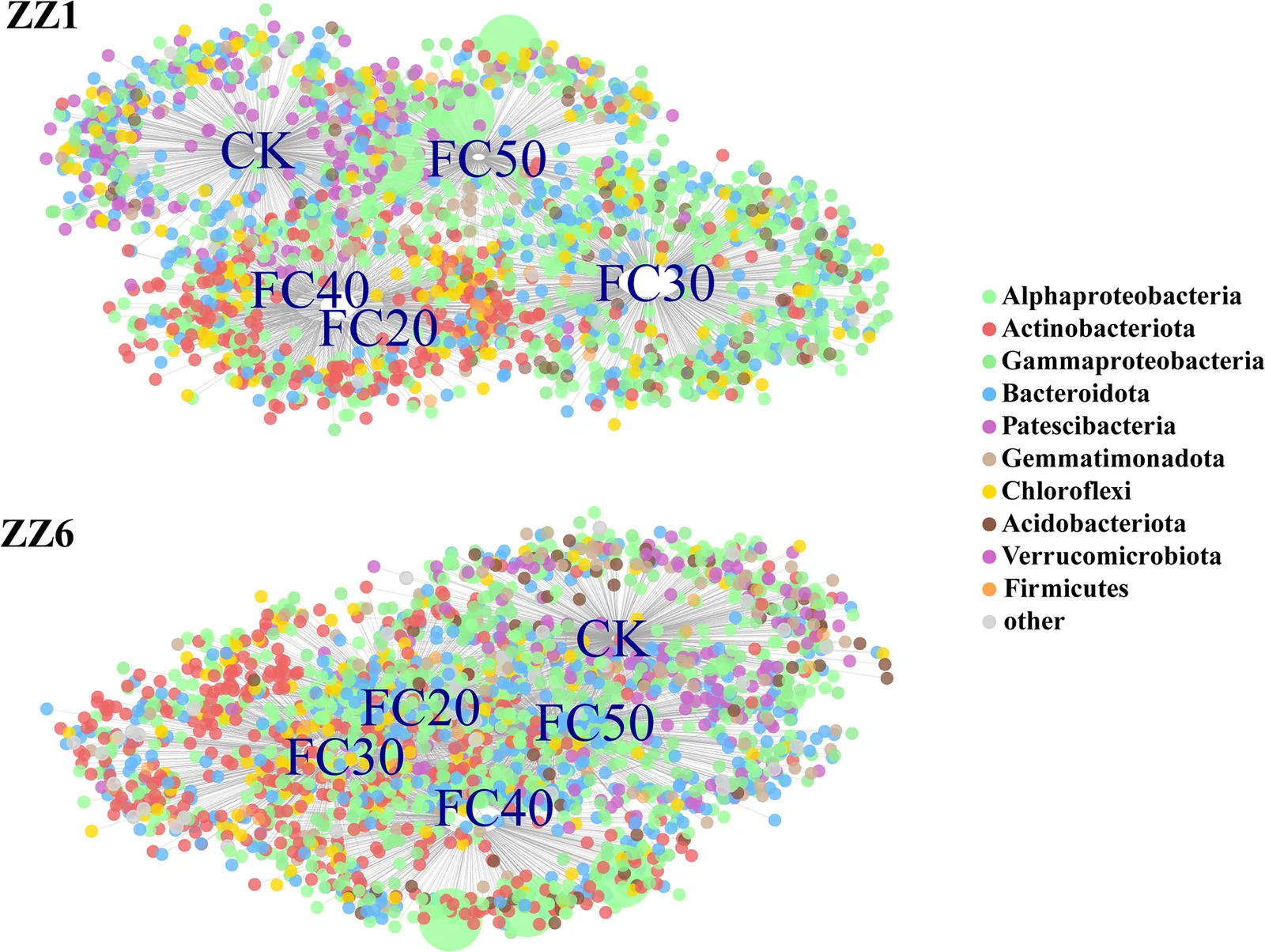
Bipartite network of rhizosphere bacterial communities reflecting OTUs under a specific drought gradient. Circles represent individual bacterial OTUs, and different colors represent the different phyla they belong to.

The clustering relationships of the responsive OTUs in the five moisture treatments were analyzed using a dichotomous network. The responsive OTUs tended to cluster together, with a stronger relationship observed among the groupings in ZZ6 compared to ZZ1 (Fig. 6). However, colonies clustered differently under different moisture stress conditions for different species. ZZ1, CK, FC50, and FC20 were surrounded by similar enrichment groups, mainly *Patescibacteria*, *Alphaproteobacteria*, *Bacteroidetes*, *Chloroflexi*, and *Acidobacteria* (Fig. 6). In contrast, FC40 overlapped with FC20 and was enriched mainly in *Actinobacteria*, *Alphaproteobacteria*, *Chloroflexi*, and *Bacteroidetes* (Fig. 6). In ZZ6, the enriched flora under CK were separated from the four water stress conditions and were mainly enriched in *Alphaproteobacteria*, *Gammaproteobacteria*, and *Patescibacteria*, whereas FC50, FC40, FC30, and FC20 had overlapping areas, and the drought fraction primarily aggregated *Actinobacteria*, *Alphaproteobacteria*, *Chloroflexi*, and *Bacteroidetes* (Fig. 6). Comparing the two varieties, the drought fraction was particularly enriched in actinomycetes, as distinguished by the control treatment.

**Fig 7:**
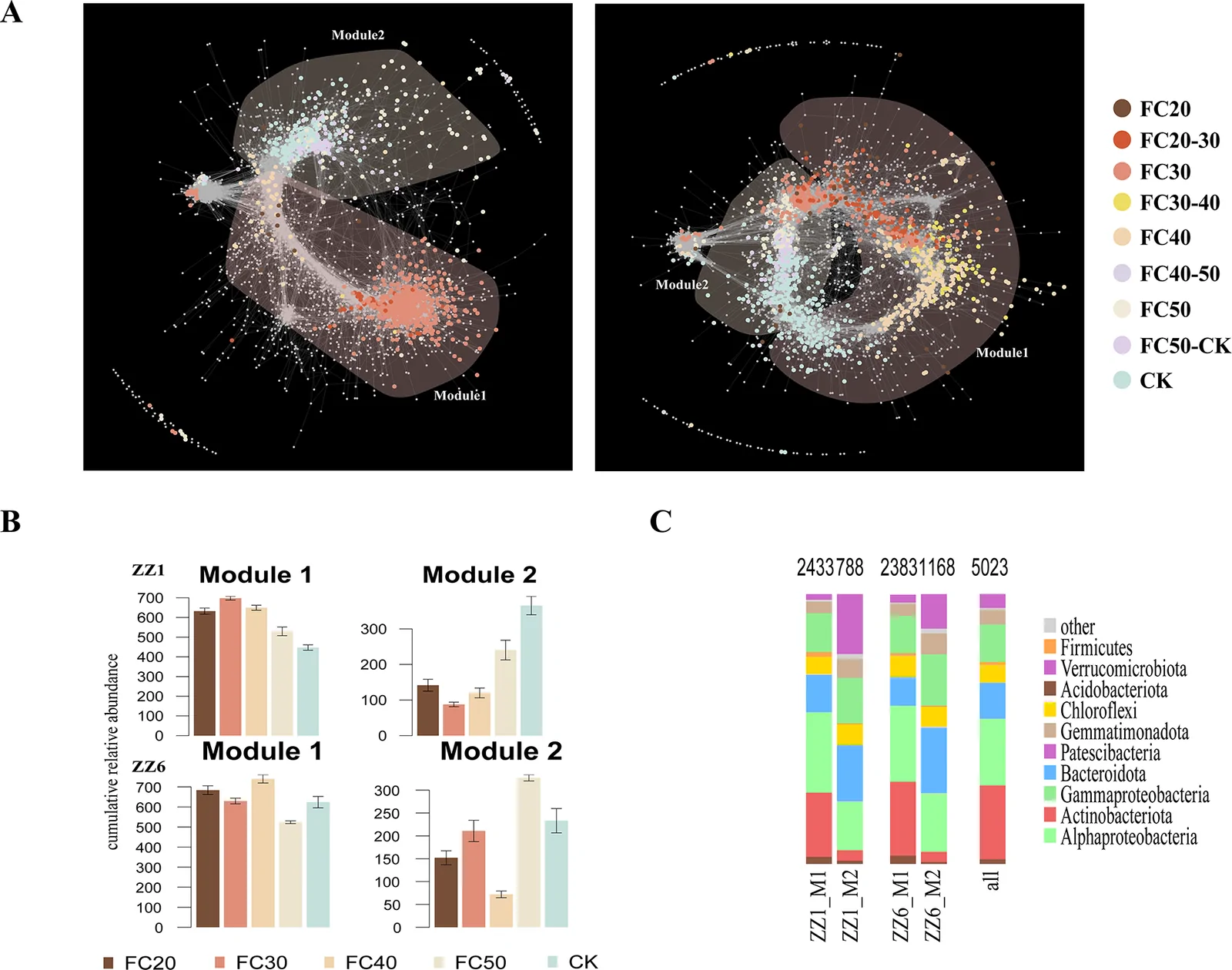
Co-occurrence patterns of sensitive OTUs. (**A**) Cooccurrence network showing the correlation of inter-rhizosphere core flora OTUs of different sugarcane cultivars under drought gradients (*ρ* >0.7, *P* < 0.001). These colored dots represent OTUs under different drought gradients. Lines between nodes indicate correlations between connected OTUs, and gray nodes indicate treatment-insensitive OTUs; shaded areas indicate network modules containing each responding OTU. (**B**) Cumulative relative abundance of bacterial OTUs in aggregated modules of different sugarcane varieties under different drought gradients. (**C**) The overall taxonomic distribution of the entire data set (the “ALL” column) is compared to the composition of the relative abundance at the class level in each module.

**Fig 8:**
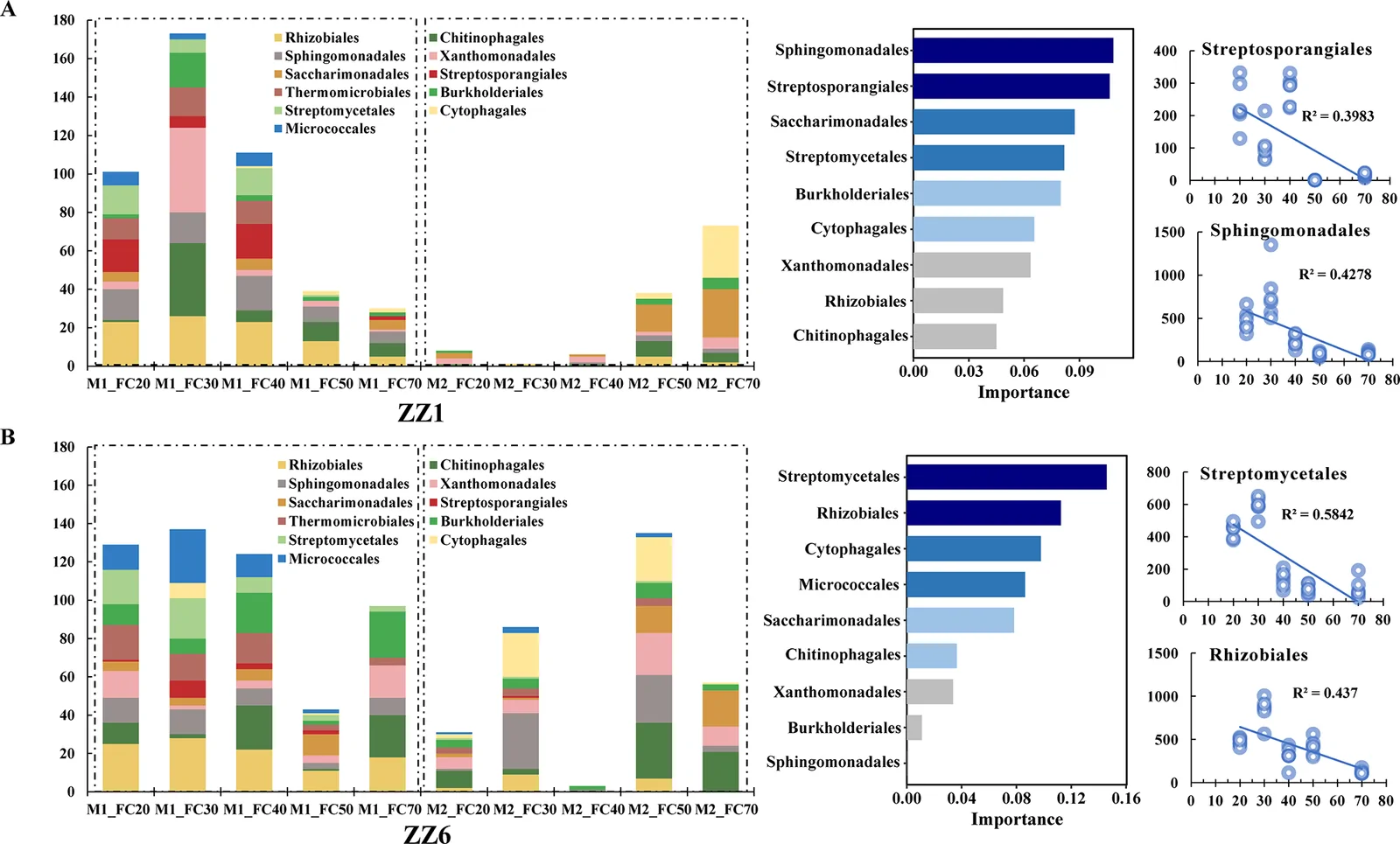
Keystone species of different species. (**A and B**) Display keystone species of different species according to the number of OTU in the gOTU co-occurrence and abundance of keystone.

Reciprocal network analysis was used to visualize the symbiotic relationships between the rhizosphere bacterial OTUs of the two species under different water treatments (Fig. 7). In the various water treatments, the rhizosphere-reactive bacteria of different sugarcane varieties were mainly concentrated in different modules (M1 and M2) (Fig. 7A). Under ZZ1, the different modules were separated, and the number of shared OTUs was reduced. In ZZ6, the modules exhibited a higher degree of aggregation and a large overlap. M1 in ZZ1 was dominated by the core flora of FC30, whereas M2 was dominated by the core flora of the CK (Fig. 7B). The highest relative abundance of OTUs that accumulated in M1 under ZZ6 at the drought level was in FC40, whereas the rhizosphere bacterial OTUs under FC50 were more dominant in M2 (Fig. 7B). Classification of OTUs in each module at the gate level revealed differences between the two species. In M1, both species were mainly dominated by *Alphaproteobacteria*. The relative abundances of *Bacteroidetes*, *Gammaproteobacteria*, *Alphaproteobacteria*, and *Verrucomicrobiota* were higher in M2 for both species, whereas the aggregation of *Verrucomicrobiota* was higher in ZZ1 than in ZZ6 (Fig. 7C). Moreover, we conducted a more detailed classification of the OTUs within each module at the genus level and observed that ZZ1 and ZZ6 exhibited similar major enrichment populations under different drought gradients in modules M1 and M2 (Fig. 8). In comparison, the core species in ZZ1 were *Streptosporangiales* (*R*² = 0.3983) and *Sphingomonadales* (*R*² = 0.4278, Fig. 8A), whereas the main groups of bacteria enriched under different drought types in ZZ6 were different from those in ZZ1, namely *Streptomycetales* (*R*² = 0.5842) and *Rhizobiales* (*R*² = 0.437, Fig. 8B).

**Fig 9:**
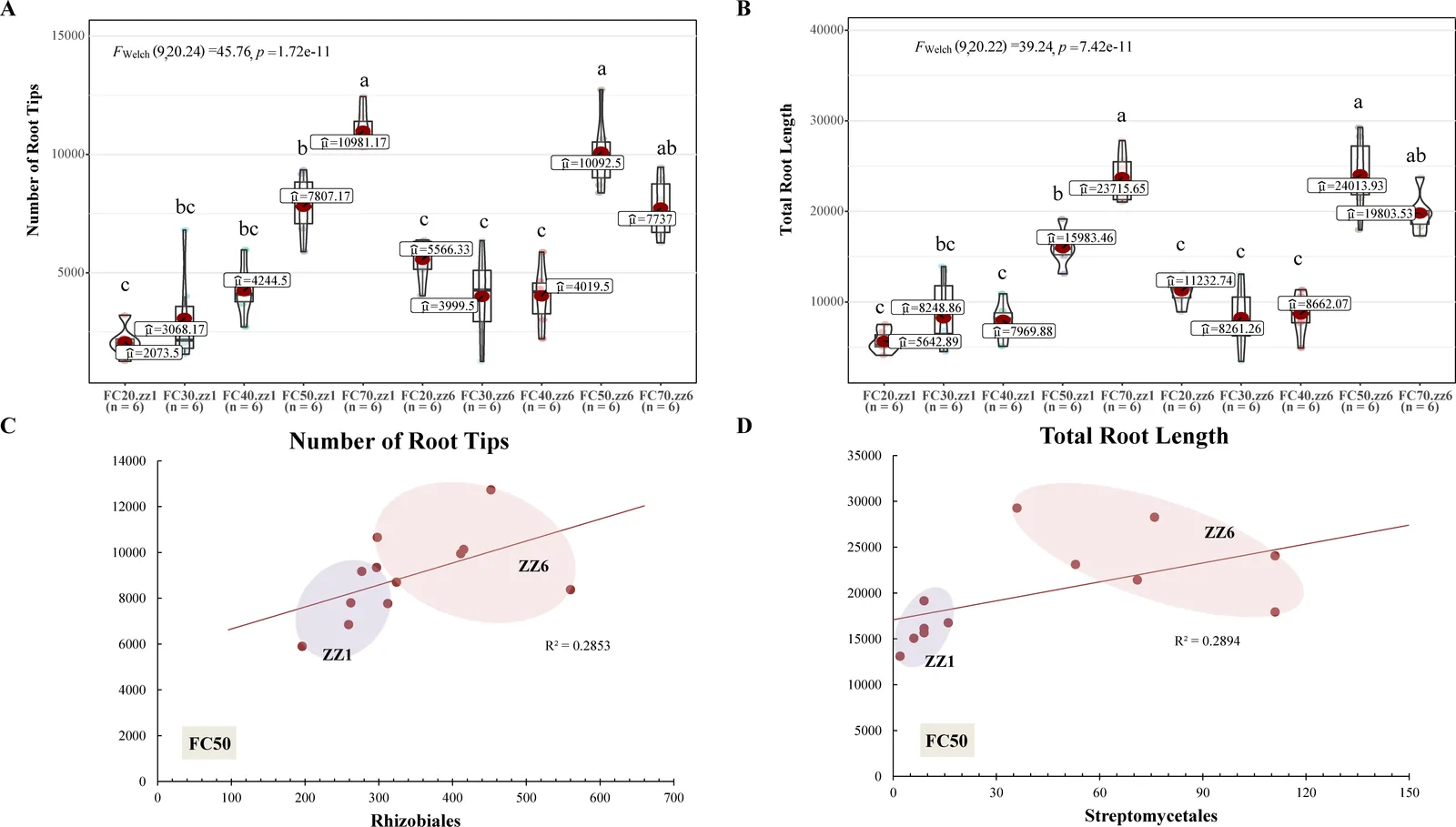
Changes in root traits under drought gradients and correlation with core strains. (**A**) Significantly different responses of root tip number of two sugarcane varieties (ZZ1 and ZZ6) under different drought gradients. (**B**) Significantly different responses of total root length of two sugarcane varieties (ZZ1 and ZZ6) under different drought gradients. (**C**) Correlation of root tip number and rhizobia between ZZ1 and ZZ6 at 50% of field water holding capacity. (**D**) Correlation between total root length and Streptomyces at 50% of field water holding capacity for ZZ1 and ZZ6. Purple ovals are ZZ1, and pink ovals are ZZ6.

Simultaneously, we analyzed the correlation between the dominant ZZ1 and ZZ6 strains with root characteristics and soil physicochemical properties under different stress levels. ZZ1 and ZZ6 showed significant differences in NRT and TRL (Fig. 9), and the two sugarcane varieties showed significant differences in FC50 (Fig. 9A and B). The core strain *Rhizobiales* of ZZ6 had a higher positive correlation with NRT than that of ZZ1 at FC50 (*R*² = 0.2853, Fig. 9C). The correlation between *Streptomycetales* enrichment and TRL was also analyzed, and ZZ6 correlated significantly more with TRL than with ZZ1 at FC50 and it was positively correlated with TRL for both Streptomyces and both species under drought stress (*R*² = 0.2894, Fig. 9D).

## DISCUSSION

### Differences in rhizosphere bacterial communities enriched by sugarcane varieties under drought gradients

A wide range of host and environmental factors influence the composition of the plant microbiome (45, 46). In this study, we investigated the impact of varying degrees of drought stress on the rhizosphere bacterial communities of two sugarcane varieties. The α-diversity of ZZ1 and ZZ6 did not differ significantly under the different drought gradients. However, ZZ6 displayed a more pronounced response to drought compared to ZZ1, which aligns with previous findings (23). The interaction between the Chao1 index of ZZ6 and root traits under drought stress highlights the influence of environmental changes and host plants on rhizosphere bacteria (47, 48). Prolonged drought conditions cause changes in the rhizosphere-associated bacterial community structure in combinations selected to adapt to abiotic stresses, improve plant root phenotypes, and thereby increase stress resistance to promote plant health and drought tolerance (47, 49). Additionally, soil properties can impact root traits by influencing nutrient and water availability, leading to variations in bacterial communities associated with the root system (48). β-Diversity analysis revealed distinct rhizosphere bacterial community compositions for ZZ1 and ZZ6 under different drought levels. The distribution of the sugarcane rhizosphere bacterial community ranged between mild and moderate to severe drought, and the community structure was influenced by the ZZ1 and ZZ6 varieties. Therefore, differences in drought resistance among sugarcane varieties affect the aggregation of rhizosphere bacterial communities, thereby improving the rhizosphere environment (50). The Mantel tests showed that ZZ6 was more closely related to environmental factors than ZZ1, particularly root area, root diameter, and soil urease. This suggests that changes in the root system and rhizosphere soil environment impact bacterial community diversity (48, 51). Plant roots obtain more water and nutrients under drought conditions by changing root phenotypes, such as root area and root diameter, to increase plant survival, whereas microorganisms favor root water and nutrients, and the root system provides a carbon source for growth (52 – 54). In turn, plant roots directly affect the soil portion of the rhizosphere by releasing exudates such as sugars and organic acids, which are sources of nutrients and vitamins used by various bacteria and stimulate the development of microbial communities (55). Similarly, soil properties can affect root traits, resulting in variations in bacterial communities associated with the root system (56, 57). Thus, we conclude that changes in root traits and soil nutrient accumulation in drought-stressed sugarcane varieties contribute to distinct bacterial community compositions, suggesting that ZZ6 may exhibit greater drought resistance than ZZ1 in response to these changes.

### Differences in core strains and interactions with plant root systems because of different sugarcane varieties

Previous studies have shown that in some sugarcane rhizospheres, microbial bacterial genera (e.g., *Arthrobacter*, *Pseudomonas*, *Microbacterium*, and *Bacillus*) can survive under drought conditions (55, 58). ZZ1 and ZZ6 differed in bacterial abundance under different levels of drought stress. Compared with ZZ1, the abundance of Streptomyces by ZZ6 increased at progressively increasing drought levels because *Streptomycetales* can improve plant adaptation to stressful conditions by producing phytohormones and biochemicals that alleviate water stress (59, 60). Additionally, significant differences were observed in the correlations between rhizosphere bacterial colonies and roots and soil in both sugarcane varieties, primarily influenced by factors such as the number of root tips, circumference, and available phosphorus. Previous studies have shown that prolonged drought stimulates the secretion of various phytohormones and osmolytes, thereby inducing morphological changes in roots and increasing stress tolerance (61). The bipartite network analysis revealed a more compact abundance of OTUs in ZZ6 compared to ZZ1, suggesting greater stability of the rhizosphere bacterial community in ZZ6 under drought conditions. Notably, further analysis of the bacterial abundance under different modules revealed that the core genera of ZZ1 and ZZ6 were different. The reason for this difference could be the difference in the genotypes and drought resistance of sugarcane varieties, where water-limiting conditions caused different responses in the two experimental varieties with varying water requirements, mainly because of the different exudates released into the soil (62 – 65). The amount and composition of root secretions produced by plants can vary depending on plant genotype, and these differences may be directly or indirectly reflected in the rhizosphere microbial community (27, 66). In conclusion, our findings demonstrate that ZZ1 and ZZ6 exhibit different core genera enriched under drought conditions, leveraging rhizosphere microorganisms to influence root and soil physicochemical properties, thereby improving drought resistance in challenging environments.

Changes in the bacterial community structure associated with the root zone of plants select assemblages adapted to abiotic stresses and improve stress resistance through metabolic activities and plant interactions, which promote plant growth and productivity (47, 67, 68). We observed that rhizosphere bacteria started to change at FC50, which could be due to the presence of mild signs of drought altering the availability of exudative sugars, organic acids, and other metabolites from plant roots that could be used for bacterial nutrition and energy, affecting the selection and enrichment of soil bacterial communities by plant roots (37, 69). Our study revealed that core strains of *Streptomycetales*, *Sphingomonadales*, and *Rhizobiales* were enriched in the rhizospheres of ZZ1 and ZZ6 under drought conditions. Previous research has shown that *Streptomycetales*, *Sphingomonadales*, and *Rhizobiales* have plant growth-promoting effects (35, 70, 71). PGPR (plant growth-promoting rhizobacteria) increase tolerance to stress by affecting phytohormone activity, alteration of root morphology, ACC deaminase activity, and accumulation of osmolytes (72, 73). The positive correlation of ZZ6-enriched *Rhizobiales* and *Streptomycetales* with number of root tips and total root length under drought conditions was significantly higher than that of ZZ1, which could be attributed to the higher abundance of *Streptomycetales* and *Rhizobiales* enriched in the rhizosphere of ZZ6 than in ZZ1. Sugarcane with strong resilience can resist drought and other stress conditions using rhizosphere microorganisms in a drought environment (32, 74). A study of the effects of drought on the microbiome found a significant correlation between the relative abundance of *Streptomycetales* in plant roots and host drought tolerance (75). *Streptomycetales* have potential benefits for host plants under drought stress, possibly through the production of phytohormones and biochemical activities that alleviate water stress (59). *Streptomycetales* have been reported to produce IAA, which results in an enhanced growth hormone response in the root tip meristem and vascular tissue, thereby enhancing the formation of lateral roots and root hairs (24, 76). This phenomenon leads to an extensive root surface of the plant contacting the soil, thereby promoting the anchoring of the plant in the soil, increasing the ability of the root system to absorb water and nutrients, and aiding plants cope with water deficits (77, 78). Nitrogen and phosphorus are crucial nutrients limiting plant growth. *Streptomycetales* and Azotobacter can mitigate drought stress in host plants by providing sufficient amounts of soluble phosphate and nitrate and enhancing the uptake of directly available nutrients by plants under stress through phosphate solubilization and nitrogen fixation capacity (28, 30). In conclusion, sugarcane begins to alter the enriched rhizosphere flora when it experiences slight drought. The predominantly enhanced *Streptomycetales* and *Rhizobiales* may affect root phenotypic changes, boosting nutrient and water intake by sugarcane under drought. We have also shown laterally that ZZ6 is more drought resistant than ZZ1.

## MATERIALS AND METHODS

### Cultivar selection and field experiment design

This study was conducted in a glass greenhouse at Guangxi University, Nanning, Guangxi, China (107°31′–108°06′ E, 22°17′–22°57′ N; 83 m a.s.l.) during the summer of 2019. The experimental site is situated in a subtropical monsoon climate zone characterized by long summers and short winters. The mean temperature, annual sunshine duration, and annual precipitation at the experimental site were 22°C, approximately 2,600 h, and 1,050‒1,300 mm, respectively. The soil used for planting was obtained from fields with a history of long-term sugarcane cultivation and exhibited the following characteristics: pH, 6.15; organic matter content, 19.47 g/kg; total nitrogen (TN) content, 100.5 g/kg; total phosphorus content, 22.4 g/kg; total potassium content, 7.11 g/kg; alkaline hydrolyzed nitrogen, 136 mg/kg; available phosphorus, 83 mg/kg; and available potassium, 77.1 mg/kg. The sugarcane varieties ZZ1 and ZZ6 selected for the experiment were obtained from the same parent, ROC25 × Yunzhe 89–7 (23), which was bred by the State Key Laboratory of Conservation and Utilization of Biological Resources in Subtropical Agriculture, Guangxi University. The potted planting method was used, and the experimental pots comprised white plastic buckets (bottom diameter, 40 cm; height, 70 cm) with holes drilled in the bottom for ventilation and drainage. Sugarcane segments with complete buds were chosen and treated with basal fertilizer to ensure consistent germination. Seedlings showing consistent growth were selected and transplanted into pots after germination. To ensure that the root system had sufficient room for growth, one plant was transplanted per pot, and each pot was subjected to the same treatment. The experiment was set up at five drought levels with soil water contents of 70% (CK), 50% (FC50), 40% (FC40), 30% (FC30), and 20% (FC20) field capacity. The sugarcane segments were grown under normal watering for 30 days to begin the drought stress exercise, which was performed by natural drought from normal soil water content to the soil water content required for different levels of the drought exercise. Samples were collected at the end of the experiment. The soil water content of each sample was measured using a soil water content measuring instrument before 6 p.m. each day, and the amount of watering required to bring it to the required water content for testing was calculated. The CK treatment received normal watering throughout the experiment to maintain optimal soil water content and served as the control.

### Soil sample collection and physicochemical analysis

Soil samples were collected from the soil attached to the surface of the sugarcane roots, and the soil immediately adjacent to the roots was identified as the rhizosphere soil. Sugarcane roots were removed intact along with the soil at the end of the drought stress period. Large external pieces of soil were carefully removed, and small amounts of soil not attached to the roots were gently shaken off. A small brush was used to clean the soil attached to the roots. Six samples were collected from each treatment group, sealed in sampling bags, and immediately refrigerated. One sample was stored at −40°C to measure the physicochemical properties of the soil, and the other at −80°C for extracting the rhizosphere microbial DNA within 24 h (74). Soil enzyme levels were measured using soil urease (S-UE), soil sucrose (S-SC), soil catalase (S-CAT), and soil acid phosphatase (S-ACP) kits (79, 80). The chemical properties of the soils were determined using the methods of previous studies (81); SOC was determined using the potassium dichromate sulfate oxidation method, TN using the semi-micro Kelvin method, and AP using the molybdenum-antimony colorimetric method. The root system was gently washed in a large bucket of water to avoid root damage. Subsequently, the roots were placed in a specialized photo box to capture images of the root system from all sides and then stored in sealed sampling bags under refrigeration. The obtained photographs were analyzed using Image J-win64 software to obtain data on root morphology and geometric conformation. Chlorophyll fluorescence was measured using a portable fluorometer (PAM-210; Walz, Germany). The measurement beam was applied to the leaves of dark-adapted plants for 30 min to measure the minimum fluorescence (*F*_0_), which was subsequently applied to determine the maximum fluorescence (*F*_*m*_). The *F*_*v*_/*F*_*m*_ value was calculated as *F*_*v*_ = *F*_*m*_ − *F*_*0*_. MDA and proline contents were determined according to the instructions provided in the microassay kits (MDA2-Y and PRO-1-Y, respectively; Komin, Suzhou, China) (82). Leaf chlorophyll content was measured using a chlorophyll meter (SPAD-502 Plus; Spectrum Technologies, Aurora, IL, United States). Three leaves from three plants in each treatment group were selected to determine the chlorophyll content. Leaf water potential was measured using a dew point water potential meter (WP4; Decagon Devices, Inc., Pullman, WA, United States) between 11:30 and 12:00 using the youngest fully expanded leaves. To determine leaf water potential, five points were selected from the tip to the base of each leaf, and the measurements were repeated on three different plants in each treatment group.

### DNA extraction, amplification, and sequencing

Microbial DNA was extracted from each fresh soil sample (1 g) according to the instructions of the E.Z.N.A. soil DNA kit (Omega Biotek, Norcross, GA, United States). Three extractions were performed for each sample, and the supernatants were combined to maximize DNA yield. The DNA concentration and purity were determined using a NanoDrop One spectrophotometer (Thermo Fisher Scientific, Waltham, MA, United States). The polymerase chain reaction (PCR) amplification of the V3-V4 region of the 16S rRNA gene was performed using 338F (5ʹ-ACTCCTACGGGAGGCAGCAG-3ʹ) and 806R (5ʹ-GGACTACHVGGGTWTCTAAT-3ʹ). The PCR mixture contained 25 µL of 2× Premix Taq (Takara Biotechnology, Dalian Co., Ltd., Dalian, China), 1 µL of each primer (10 M), and 3 µL of DNA (20 ng/µL) template for a total volume of 50 µL. Thermal cycling was performed in a thermal cycler (Biometra, Goettingen, Germany) under the following conditions: 5 min initial denaturation at 94°C, followed by 35 cycles of denaturation at 94°C for 30 s, annealing at 52°C for 30 s, extension at 72°C for 30 s, and a final extension at 72°C for 10 min. Illumina TruSeq DNA sample preparation kits (Illumina, San Diego, CA, United States) were used to construct DNA libraries. The PCR products were sequenced by Magigene Technology (Guangzhou, China) using the Illumina HiSeq 2,500 platform, resulting in 250 bp paired-end reads. The 16S rRNA gene sequences obtained in this study were stored in the National Center for Biotechnology Information Sequence Read Archive (registration number: PRJNA858879).

### Statistical and bioinformatics analysis

Quality filtering on the paired-end raw reads was performed under specific filtering conditions according to the Trimmomatic (V0.33) quality-controlled process. Paired-end clean reads were merged using FLASH (V1.2.11) according to the relationship of the overlap between the paired-end reads; when at least 10 of the reads overlap the read generated from the opposite end of the same DNA fragment, the maximum allowable error ratio of the overlap region of 0.1 and the spliced sequences were called Raw Tags (83). Raw tag sequences were assigned to unique barcodes and primers using Mothur (V1.35.1) software to obtain clean reads (84). Zero-radius operational taxonomic units (ZOTUs) and their representative sequences were generated using UNOISE3 non-clustering denoising (85). Sequences with an abundance of 12 were retained, and low abundance sequences were discarded due to the higher likelihood of containing errors (86). And using the “otutab” command in USEARCH (v10), the representative sequences of ZOTUs were mapped to the clean raw reads to generate the OTU table. Finally, the OTU representative sequences were classified in the SILVA database (V132) to obtain species taxonomic annotation results for the species (87).

Alpha diversity (α-diversity) was assessed using the Chao1 and Shannon diversity indices to obtain the richness and diversity of species in environmental communities, respectively. Correlations between α-diversity and soil properties were shown using the “corrplot” package in R (V3.6.3) (88). Beta diversity (β-diversity) and phylogenetic communities were compared using weighted and unweighted UniFrac distance matrices. Mantel tests were used to investigate the relationships between β-diversity and environmental factors and between enriched bacterial OTUs and environmental factors. Taxonomic composition was determined based on the relative abundance of the dominant clades. The “alluvial” and “ggplot” packages in R (V.3.6) (89) were used to assess changes in the relative abundance of bacterial communities in each compartment. dbRDA was used to determine the relationships among root characteristics, soil characteristics, and soil bacterial OTUs. Mantel testing, principal coordinate analysis, and dbRDA were performed using the “vegan” package in R (V3.6) (90).

### Bipartite and co-occurrence networks

Bipartite network visualization was used for the metric analysis of significant water stress patterns (*P* < 0.05), and TMM normalization was used for all networks. Spearman’s correlation analysis was also performed to determine the correlations between OTUs. In addition, Spearman’s correlation analysis was used to assess the association between environmental factors and rhizosphere bacteria to determine topological network properties. These included the total number of network nodes (representing OTUs), total number of edges (connections between nodes indicate significant positive correlations between OTUs), and degree of co-occurrence (number of direct correlations with nodes). We then constructed meta-networks to visualize the correlations between bacteria in the soil and root communities. For this purpose, we added TMM-normalized CPM bacterial counts to the OTU tables of the soil and root communities and performed Spearman rank correlations between all OTU pairs. Network properties were summarized and analyzed to identify network modules and the community structure of each plant unit network (91). Microbial taxa that repeatedly coexist in microbial co-occurrence networks are considered ecologically important and play key roles in the microbiome (92, 93). In each water stress model, OTUs with network node degree values in the top 1% were identified as crucial meta network OTUs. We prioritized this simple definition over another complex approach because both definitions reveal the same key OTUs. In addition, the correlations between the modules and soil environmental factors were investigated using the Mantel test (94).

## Data Availability

The data sets generated for this study can be acquired from NCBI PRJNA858879.
